# Sleep and Mental Health in Truck Drivers: Descriptive Review of the Current Evidence and Proposal of Strategies for Primary Prevention

**DOI:** 10.3390/ijerph15091852

**Published:** 2018-08-27

**Authors:** Sergio Garbarino, Ottavia Guglielmi, Walter G Sannita, Nicola Magnavita, Paola Lanteri

**Affiliations:** 1 Department of Neuroscience, Rehabilitation, Ophthalmology, Genetics and Maternal/Child Sciences, University of Genoa, Polyclinic Hospital San Martino IRCCS, 16132 Genoa, Italy; ottavia.guglielmi@gmail.com (O.G.); wgs@dism.unige.it (W.G.S.); 2 Institute of Public Health, Università Cattolica del Sacro Cuore, 00168 Roma, Italy; nicolamagnavita@gmail.com; 3 Neurophysiology Center, Department of Medical and Surgery Neuroscience, Rehabilitation–Continuity of Care, IRCCS Institute G. Gaslini, 16147 Genoa, Italy; paolalanteri@gaslini.org

**Keywords:** truck driver, sleep, sleep disorder, mental health, depression, anxiety, prevention, road safety

## Abstract

*Background*: Professional truck drivers (TDs) are exposed to stressful working (and living) conditions and are vulnerable. They report physical and mental health problems and psychological distress more frequently than the general population and their problems can affect safety on the roads. Actions to improve TDs’ health and reduce the risks of (co-)morbidity or unsafe driving are imperative. *Methods*: The published studies dealing with the TDs’ sleep habits and mental health were reviewed to define the scenario and organize the preventive strategies proposed thus far. *Results:* Awareness among TDs of the high risk for health and safety due to (often co-existing) untreated sleep and mental health problems is critical. Alcohol and prescribed or illicit drugs are often misused to compensate for depression, anxiety, job strain, fatigue, and social isolation. Polypharmacy and dependence increase the chance of unsafe behaviors on the road. The TDs’ access to healthcare services is scant, and participation in industry-sponsored wellness programs is limited. *Conclusions*: Primary prevention is a first unavoidable step to deal with sleep and mental health problems. Educational programs, online support and tele-health assessment/monitoring would help improve the well-being, safety and health of professional TDs and increase safety on the road.

## 1. Introduction

Professional truck drivers (TDs) work in stressful conditions that favor unhealthy lifestyles and medical disorders. Their overall health, and especially their mental health, is very often worse than the general population as a consequence of long driving shifts, disrupted sleep patterns, chronic fatigue, social isolation, compelling service duties, delivery urgency, job strain, low rewards, and unsystematic medical control [1,2,3,4,5,6,7,8,9,10]. Indeed TDs, especially those who drive long-haul routes, face a multitude of mental health-related risks attributed to the transportation environment [11] such as, in addition to those indicated above: spending many consecutive days away from home and family, constant time pressure due to demands of “just in time” delivery requirements (high demand and low control), stimulants, alcohol and drug use [12]. The trucking industry work environment emphasizes stoicism, independence and emotional control which favors symptoms of low mood or distress (anger, risk taking, memory and concentration deficit, anxiety, depression, insomnia) [13]. The prevalence of minor psychiatric disorders was 6.1% [14], of depression 13.6% [15], anxiety 7.9% [16].

Their body mass index (BMI) is on the average higher than in the general population and other occupational conditions; also higher are the risks of chronic diseases (particularly metabolic syndrome, diabetes mellitus, cardiovascular disorders and premature heart disorders) [4,9,17,18,19]. Obesity and overweight in the food addicted is most likely to be accompanied by depressed mood with associated negative self-evaluation and a sense of despair and futility around the elusive goal of weight loss [20]. TDs’ health usually worsens during life and the incidence of co-morbidity appears to be increasing [21]. The impact of unhealthy lifestyles and occupational diseases seems severe: male truck drivers in USA have a shorter life expectancy (63 years and 55.7 years for unionised and independent TDs, respectively) compared to the general population (75.1 years) [11].

Safety on the road compares to health as a major issue, with some of the TDs problems potentially affecting road safety [1]. Adequate levels of vigilance and focused attention are in fact necessary when driving and sleep disorders or inadequate sleeping become crucial in this respect because of the resulting excessive sleepiness during the day. Sleep disorders are often co-morbid: for instance, obesity and the metabolic syndrome are strongly associated with sleep disorders such as the obstructive sleep apnea (OSA) [22], which *per se* causes excessive daytime sleepiness and is associated with a 2-to-8 times increased risk of accidents [23,24,25,26]. The risk increases further (55%) among obese drivers of heavy commercial vehicles compared with TDs with normal weight [3]. The associated use of medications (either prescribed or self-administered), alcohol and/or amphetamines increase the risk of accidents [27,28].

Poor sleep/sleep disruption is today a recognized problem in medicine and healthcare [29]. The sleep-wake cycle is a major expression of the physiological mechanisms underlying the circadian rhythm (process C) and homeostatic process (process S); this is essential to the homeostatic processes that guarantees survival, adaptation, efficient and reliable action in everyday’s life, and wellbeing [30]. Sleep characteristics such as duration, quality, timing and variability have been associated with a wide range of health outcomes, while the time congruency or misalignment with the 24 h circadian cycles have not been investigated systematically [31]. Also, as a consequence of this, robust theoretical constructs for the observed association between sleep/sleep disturbances and mental health are not available. In particular, the potential relevance in occupational health has been largely overlooked thus far [32].

Purpose of this paper was to provide a descriptive review of the relationship between sleep and mental health and its impact on TDs. A detailed scenario would also help identify preventive strategies for the TDs’ health and wellbeing and devise countermeasures to control the potential risks on TDs and on the general population.

## 2. Sleep-Mental Health interplay

### 2.1. Psychiatric Disorders

Psychiatric disorders and disordered sleep-wakefulness or circadian rhythms are frequently associated, often with bidirectional relationship as in the case of insomnia and depression [33,34,35]. Insomnia occurs in over 90% of patients with clinical depression [36] and is an established risk factor for depression (odds ratio for developing depression: 6.2) and relapsed depression in treated patients [37]. Subjects with untreated insomnia have higher prevalence of major depression or anxiety than non-insomniacs (odds ratio = 39.8) [38]; in turn, depression is a risk factor for insomnia (odds ratio: 6.7) [39]. High perceived stress, psychological distress, anxiety and depression are significant predictors of poor sleep quality and persistent insomnia [40,41] and can contribute to insomnia-related hyperarousal thus perpetuating chronic insomnia [42,43]. Insomnia is the most common sleep disturbance associated with anxiety disorders [34] and poor sleep quality is frequent in adults with anxiety disorders [44], who experience disturbances in the sleep-onset or sleep maintenance and insomnia. The association of insomnia and OSA is frequent (29.2%) and stands as a cumulative risk factor for cardiovascular diseases [45] while favoring arousal-inducing sleep-related behaviors, higher levels of pre-sleep arousal, anxiety, and depression more than OSA alone [46].

### 2.2. Substances of Abuse

Sleep is also impaired by pharmacological abuse/misuse and subjects using neuroactive drugs frequently experience inadequate sleeping and its detrimental effects [36]. Neuroactive substances act on receptor- and neurotransmitter systems which also mediate in sleep regulation. Insomnia is three times more frequent in subjects with drug abuse/misuse [47,48] and in turn predisposes to the abuse of sedative-hypnotic agents with resulting excessive daytime sleepiness (EDS) or pharmacological tolerance. Alcohol is the most common sleep inducer; with habitual use, sleep induction is substituted by disruption of its physiological organization and continuity. Insomnia is reported by 36–72% of alcohol-users and may persist weeks to months after beginning of abstinence [49] and insomniacs report alcohol-related problems twice as often as normal sleepers; subjects abusing sedative or hypnotic drugs further complicate their sleep disturbances. Drugs like cocaine can induce psychosis and insomnia. Chronic usage may lead to tolerance, and EDS, sluggishness, ataxia, slurred speech, and visual-motor problems with late-afternoon restlessness and nervousness at increased doses [34]. Sleep problems during childhood (3–5 years) are possible early markers for the increased risk of abusing alcohol, marijuana, or illicit drugs later in life [50]. Individuals using/abusing central nervous system stimulants like phenylethylamines (amphetamine, ephedrine), cocaine, thyroid hormone, and xanthine derivatives (caffeine or theophylline in excessive doses) have sustained periods of total sleep suppression, often followed by periods of severe hypersomnia. These compounds are frequently associated with hyperactivity eventually worsening into hypomania, garrulousness, paranoid ideation, and compulsive behaviors [34]; withdrawal may include dysphoria, fatigue, vivid and unpleasant dreams, insomnia or hypersomnia, increased appetite, and psychomotor retardation. Increased arousal is an established key factor for chronic insomnia; both conditions induce high aminergic output and medications potentiating the central aminergic balance or conditions decreasing inhibitory neurotransmission can cause insomnia.

## 3. Review of the Impact of Sleep-Mental Health Interplay on Truck Drivers

The interplay of sleep and mental health problems TDs has received limited attention by the scientific community, we tried to critically review the current knowledge in the literature to assess the state of art of this argument. A systematic review and a meta-analysis have been proved to be impracticable due to the small number of papers on the issue and the differences among countries and regulations. In addition to this, sleep, sleep disorders and mental health have been assessed to some extent differently, in different studies. However, we examined different databases such as PubMed, Medline, and EMBASE. Search terms included truck drivers and sleep habits, health, sleep disorders, sleep apnea, sleepiness, mental health, psychiatric disorders, depression, anxiety, stress. An updated search in the scientific literature found only eight papers published to date (April 2018) that addressed both sleep habits and mental health at the same time. Three reported on Italian, two on North American, two on Brazilian, and one on Australian truckers, for a total sample size of 87,261 male and 3887 female drivers [8,51,52,53,54,55,56,57]. Some comparison across studies is nevertheless possible with a descriptive review. The features of the eight selected papers about sleep and mental health in truck drivers are summarized in Table 1 and Table 2.

### 3.1. Sleep Disorders

Unspecified sleep disorders were reported by 20% to 28.6% of truck drivers, insomnia by 27.5%, OSA by 25.8–51%. EDS The mean average sleep duration was slightly shorter than the usually recommended 7 h in two studies [8,55] and less than 6 hrs. in 17.3% of subjects in another study [55], with very short sleeping (<5 h/night) in 22.2% and 7.7% of cases. Prevalence of OSA was significantly higher among insomniacs, who also had a higher prevalence of cardiovascular diseases, diabetes, depression (13.8% vs. 7.4%), and respiratory disorders, an almost double risk of motor vehicle accidents (MVAs) (OR: 1.82, 95% CI: 1.33–2.49), and an over three-fold increased risk of near-miss accidents (NMAs) (OR: 3.35, 95% CI: 2.06–5.45) than other drivers.

### 3.2. Obesity and Co-Morbidity

A risk factor for co-morbidity as important as diabetes, hypertension and OSA [58,59], obesity can also interfere with safe driving. It has proven associated with the risk of road accidents, with a more than double crash rate compared to non-obese drivers [3,60,61]. All published papers but one [55] assessed BMI: the mean BMI was always higher than 26 kg/m^2^ among TDs reporting sudden sleep onset at the wheel or involved in road accidents, particularly in drivers working in shifts. BMI was 30 or higher in over half of studied TDs. One study [56] reported 83.4% of over-weight or obese drivers (BMI 30 or more (obese) in 53.4%, BMI 25–29.99 (overweight] in 30%). One study [57] reported a BMI of 30 kg/m^2^ or more in 53.2% of subjects, with the percentages of BMI 35.0 kg/m^2^ or more and 40.0 kg/m^2^ or more being 26.6% and 12.1%, respectively. One study [57] indicated consistent trends of association of high blood pressure (OR = 3.61), diabetes mellitus (OR = 4.15), and sleep disorders (OR = 5.49) among obese drivers as compared to normal weight drivers. After adjustment for age and sex, overweight and obese drivers were more likely to have abnormal examination findings for general appearance, heart, mouth, abdomen, and vascular system as compared to normal weight drivers. Mean BMI increased from 30.6 kg/m^2^ to 32.6 kg/m^2^ over three years. 

Obesity has been associated also to a higher incidence of psychiatric symptoms and reportedly co-exists in TDs with poor sleep quality which, in turn, is associated with psychological distress [62]. However, 1.3% of TDs rated their overall mental health as poor or very poor only in one study [56], whereas the reported incidence of self-rated depression, feeling of loneliness, anxiety, use/misuse of neuroactive drugs was higher: 27.9% reported previous or current feelings of loneliness, 26.9% depression, and 20.6% sleep problems. In addition, 18.9% reported experiencing chronic fatigue, 16.8% drug abuse/misuse, 14.5% anxiety disorders, and 13% “other emotional problems” (feeling tired or having low energy, trouble sleeping, and headaches, and the like). When asked about specific symptoms of anxiety over the past four weeks, 47.2% reported becoming agitated easily, 43.8% feeling fidgety, 41.8% having difficulties in winding down, 37.5% having problems when trying to relax, 35.7% over-reacting; 35.4% feeling nervous, and 28.2% being poorly tolerant. Depression was reported by 26.9% of TDs investigated in this study, at variance with percentage of 9.2%, 7.5% and 1.8% in other studies [51,53,54], and 12% vs. 3% if sample was subdivided between drivers involved in police-reported crashs (cases) and those not involved in crashes in the past year [54]. When combining psychiatric symptoms together, 6.6% of TDs suffered psychiatric disorders in a sample study of 514 male [55] and 11.1% suffered emotional stress. Distress was perceived in 19.7% [52].

### 3.3. Substance Abuse/Misuse

Drinking alcohol proved to be common among TDs; 24.2 to 77.0% of subjects reported having used alcohol within the previous year [53,55]. Over one third (33.5%) reported using alcohol at least once a month, 11.7% drinking 1 or 2 drinks per day, and 24.7% having 6 or more drinks per night on occasions [56]. Six percent of TDs were reportedly unable to stop drinking, 0.9% reported drinking first thing in the morning, 4.1% feeling guilty over drinking, and 4.7% having memory problems after drinking. Drinking alcohol was more frequent among amphetamine users [55]. About 4.7% of TDs have reportedly used at least one illegal drug (other than amphetamines) within the previous year [55]. Among illicit substances, cannabis was the drug of choice, with opioids (2.5%) and cocaine (2.2%) ranking second and third preferred drugs. Four percent reported that their drug use affected their emotions, 2.8% had a strong urge to use drugs, 1.3% used more drugs than they intended to, and 2.2% used drugs for their specific effects. Only 1.8% reported using drugs for anxiety and depression [53]. About 65.4% of TDs driving on day-shifts consumed alcohol, 11.5% smoked cigarettes; the corresponding figures were 54.8% and 16.1% for workers in irregular-shifts [8].

### 3.4. Safety and Risk of Road Accidents

Only three of the eight published papers [51,53,54] included in the current analysis have investigated the role played by sleep and/or mental health in safety as risk factors for road accidents (Table 2). Both sleep and mental problems induce an increase in OR for driving accidents: OR for driving accidents or near-miss accidents for sleep disorders is 1.82 and 3.35 respectively [51] for insomnia and 3.42 [54] for OSA, OR for depression is 6.05 [54]. OR for TDs not completed fatigue management is 6.05 [54], OR for sudden-onset sleepiness at the wheel is 5.22 in TDs older than 55 years old, 2.89 if driving more than 50,000 km per year, 2.97 in TDs with Chalder Fatigue Questionnaire score > 11 [53].

### 3.5. Medical Care

In contrast with the incidence of reported psychiatric symptoms and sleep disturbances, the majority of TDs reported of not receiving adequate professional attention and treatment for their problems [56]. Only 8.4% reported receiving prescribed medication for mental health-related problems (antidepressant therapy in 7.8%). TDs blamed: their not being in need of a doctor (10.8%) or their unpredictable working schedule (23.4%), the lack of health insurances (16.1%), the inadequate servicing by the Department of Transportation (10.1%), their neither trusting nor liking doctors and not believing in medicine (5.1%); their being unable to afford medical care despite health insurance (4.4%), the inconvenience or inaccessibility of health care (4.1%); their having multiple housing locations (3.8%), not knowing where to go (3.5%), language problems (0.6%), and unavailability of regular doctor (0.3%).Left untreated mental and sleep health related problems can potentially lead to an increase in risk road accidents and risk-taking behaviors as substance misuse which in turn can lead to road accidents, too [26,54,56]. A significantly increased risk of crash involvement can have potential ramification for public health. More rigorous screening and subsequent treatment of OSA, insomnia and depression by clinicians as well as compulsory fatigue management training may reduce crashes among heavy vehicle drivers in order to improve health and safety on the road.

## 4. Possible Strategies for Prevention

Professional truck driving is a major cause for occupational stress, sleeping on irregular schedules, subjective fatigue, low access to and limited attention from public or private healthcare, and scanty opportunities for social support. Workers in this large and growing occupational segment are at risk for a range of occupation-induced conditions in which the detrimental combination of mental health problems and sleep disorders may impact severely [11]. TDs often overestimate their overall health condition and underestimate their problems, but readily describe particular mental health-related problems, often exacerbated by the stressful occupational environment. The studies reviewed in this paper set forth suggestions for a congruent and potentially effective prevention. In order to have chances of success, an *ad hoc* strategy should include:

1. Occupational health physician:
  a) Implemented collective educational projects included in a comprehensive health promotion program by occupational health physician for workers and employer, specifically purported to:
    1. favor healthy lifestyles, promote proper diet and physical activity (including conscious and safe sex).
    2. increase the awareness of the deleterious effects and health risks due to obesity and over-weight.
    3. advise (and possibly favor) proper sleep habits, sleep health, working schedules giving opportunities for adequate sleep or compensation for working at night or in shifts (sleep hygiene, naps).
    4. inform in full detail about the relationship between sleep disorders and psychiatric mental problems.
    5. promote a growing awareness of the epidemiology of mental disorders and mental health-related problems and of their burden on individuals, society, family and work environment.
    6. disseminate detailed information about the health risks and damage of alcohol, misused therapeutic compounds, and illegal drugs and discourage their misuse/abuse [63].
    7. management of safety at the workplace for reducing of risk of injury and road accidents [63].
    8. provide through education and training appropriate tools to countermeasure compulsory fatigue and manage sleep [54,56].
  b) into current health surveillance activities:
    1. screening of sleeping disorders (EDS, OSA and insomnia) and intervention to determine poor sleep quality can be incorporated into current health surveillance activities without a significant increase in medical costs or lost of medical time [64,65].
    2. identify drivers (especially with limited seniority) abusing alcohol or making use of amphetamines. Drivers with health problems due to amphetamine or chronic alcohol should be referred to specialized programs providing medication (if needed), psychological counseling/support, and rehabilitation purported to decrease the risks of relapsing and to improve safety on the road [55,66].
    3. screening of mental problems and access to appropriate health services.
    4. control over the condition predisposing risks of injury [63] and number of errors and accidents.
2. Employers:
  a) pragmatic action to improve the working conditions that can cause health problems (optimize the work organization, avoid long working hours and irregular work schedules, guarantee time at home), adequate and safe cabin for sleeping
  b) make paramedical treatment available to help reduce/compensate for the gap in health care access that is common for long-haul truckers.
  c) promote and guarantee online support for TDs and remote tele-health assessments and monitoring.
3. Government and law:
  a) guarantee law enforcement about law on working time, truck stops or rest areas with facilities as park-like areas, fuel stations, public toilets, restaurants with healthy food, and dump and fill stations for recreational vehicles access
  b) limit or restrain (at least temporarily) the certifications (guide license) of TD with mental and sleep problems not treated especially if other medical condition coexists.

## 5. Discussion

The small number of identified papers *per se* testifies how the research undertaken thus far has been unsystematic. The published papers on the health and sleep patterns of TDs nevertheless confirmed how their work environment is linked to poorer health. Sleep disorders, short sleep duration, OSA, and insomnia were reported in significant proportions among TDs. The prevalence of OSA was higher in insomniac patients who also had a significantly higher prevalence of cardiovascular diseases, diabetes, depression (13.8% vs. 7.4%), and respiratory disorders. Insomniac TDs had an almost two-fold risk of motor vehicle accidents (MVAs) and a more than three-fold increased risk of near-miss accidents (NMAs) than other drivers. All authors confirm the high prevalence among truck drivers of over-weight or obesity, often associated to sleep disorders and increased incidence or worsening of psychiatric symptoms. Mental health-related problems (e.g., depression, loneliness, anxiety, substance problem) proved more difficult to assess than sleep problems, but proved common when investigated with accuracy.

Health, psychiatric and sleep problems are usually under-reported, possibly due to the need to keep the certifications required to work. Obese drivers appear to be cautious reporting, particularly multiple conditions [57]. In general, TDs appear to be insufficiently aware of the high risk for health and safety of untreated sleep and mental health problems. Nor are they conscious of the problems related to the use of illicit drugs or misuse of prescribed drugs to alleviate the symptoms of depression, anxiety, job strain, fatigue, and social isolation [67]. Neuroactive drugs (amphetamines for instance) favor improper behaviors on the road by an estimated 78% and increase the risk of accidents [68]. Brodie et al. [69] found amphetamine catabolites in the blood of one every six TDs died in road accidents. Polydrug use is also frequent and Williamson et al. [70] showed that TDs making use of stimulants violate traffic laws more often than controls and especially while at work. Combinations of medical conditions and/or drug use/misuse/abuse, alcoholic beverages and amphetamines increase the risks of traffic accidents [27,28].

The majority of TDs participating in the study reported never receiving professional treatment. If untreated, the problems they reported increase risk-taking behaviors, including unprotected sex with multiple partners or with sex professionals, or substance misuse, which in turn can lead to road accidents—all of which has a fallout on public safety and therefore on public health [56]. The truckers’ access to healthcare services is unexpectedly scant [71], and participation in industry-sponsored healthcare programs is limited [72]. Implementation of educational programs, particularly targeting obese drivers and promoting increased awareness of the deleterious effects of alcohol consumption, fatigue, mental health and sleep problems while driving, may help improve the professional drivers’ well- being as well as road safety and public health by reducing road accidents among this work category.

This review has several limitations due to the small number of identified papers, the differences among countries and regulations, so it is a descriptive and not a systematic review. In addition, sleep, sleep disorders and mental health have been assessed to some extent differently in different studies examined. The aims of these eight studies were very different and no one had the goal to study the interaction between the two principal fields of our investigation (mental and sleep health in TDs). 

Further study on relationship between both sleep problems and mental health in TDs and their impact on well-being, health and safety is needed to identify attributable proportions and relationships between these factors. An interventional study to impact the relationship between sleep problems, mental health, obesity, drug use and road accidents is also needed.

## 6. Conclusions

TDs around the world operate in a stressor-filled environment that exerts substantial adverse influence on drivers’ physical health and well-being, with access to healthcare services unexpectedly hindered despite the stringent governmental and corporate regulations. TDs are vulnerable to a variety of health risks and are also a medically underserved population. Government, industry and healthcare providers are faced with the challenge of keeping the TDs sector profitable and competitive while taking care of drivers’ health and public safety [7]. Primary prevention is a first step, and education the first pragmatic approach to a large-scale action with *ad hoc* programs, online support and tele-health assessment to identify, monitor and treat important elements (sleep disturbances, excessive daytime sleepiness, loneliness, depression and anxiety, control of obesity, alcohol consumption, drug abuse) and provide personalized medical care while avoiding self-medication.

## Figures and Tables

**Table 1:** Data summaries of the 8 published papers included in the current analysis.

Author Year—(Country)	Sample Size/Type	Type of Study	Methods	Sleep Habits and Disorders	Mental/Psychological Disorders	BMI	Alcohol	Substance Use	Caffeine	Smokers
**de Oliveira et al., 2015—Brazil) [55]**	514 males/TDs (36.7 ± 7.8 years)	cross-sectional	Self-administered questionnaire	Bad sleep quality: 56.0% (moderate to serious EDS in 33.7%)	Emotional stress: 11.1% Psychiatric disorders: 6.6%	nd	77.0%	Amphetamine: 29.0% at least one illegal drug—other than amphetamines: 4.7%,	nd	nd
**Garbarino et al., 2017—(Italy) [51]**	949 males/TDs (44.3 ± 10 years)	cross-sectional	Medical examinations, semi-structured interview, questionnaire, Berlin questionnaire, PSQI, ESS.	6.8 h average for sleeping hours short sleep duration (<6 h): 17.3% insomnia: 27.5% suspected OSA: 25.8%	Depression: 9.2%	28.06 ± 4.7	nd	nd	3.2 ± 2 cups of coffee/day	8.13 ± 12.03 number of cigarettes/day
**Guglielmi et al., 2018—(Italy) [52]**	526 males/TDs (45.9 ± 9.4 years)	cross-sectional	Self-administered questionnaires; STOP-Bang, PSQI, ESS, GHQ-12	Bad sleep quality: 17.3% ES: 8.9% at risk for OSA: 51.1%	Distress: 19.7%	26.8 ± 3.3	nd	nd	nd	nd
**Meuleners et al., 2015—(Australia) [54]**	200 males/long distance heavy vehicle drivers (45.1 ± 9.5 years)	case-control (involved or not in police-reported crashes)	Interviewer-administered questionnaire and sleep monitoring (Flow Wizard nasal pressure transducer)	OSA: 23% Control 31% Cases	Depression: 3% Control 12% Cases	29.2 SD 4.8 Control 30.6 SD 5.5 Cases	nd	nd	18% Control 15% Cases	46% Control 49% Cases
**Rosso et al., 2016—Italy) [53]**	355/ TDs (gender was not required in the questionnaire) (42.7 ± 9.7 years)	cross-sectional	Database of High Risk Professional Driver Study. CFQ, AUDIT	EDS: 48%	1.8% used drugs (anxiety and depression)	45% BMI 25–30 21.4% BMI > 30	24.2%	nd	3.3 ± 2.3 cups of coffee/day	40.1%
**Shattell et al., 2012—(USA) [56]**	316 males/TDs (44.2 ± 10.7 years)	cross-sectional descriptive design.	Self-administered 82-item HEATS	Chronic sleep disturbances: 20.6%	Loneliness: 27.9% Depression: 26.9% Anxiety: 14.5% Other emotional problems: 13%	53.4% BMI ≥ 30 30% BMI 25–29.99 16.6% BMI < 25	62% (6% reportedly unable to stop drinking)	Cannabis: 3.4% Opioids: 2.5% Cocaine: 2.2% Stimulants: 1.9% Sedatives: 1.8% Chroming/Huffing: 0.6% Hallucinogens: 0.3% Others: 2.2%	nd	nd
**Thiese et al., 2015—(USA) [57]**	88,246/over-the-road or long-haul drivers (46.0 ± 10.4 years) Males: 84,359 (95.6%), Females: 3887 (4.4%)	cross-sectional	Database RoadReady, Inc (web-based platform for commercial driver medical examinations)	OR = 5.49 sleep disorders BMI >30 OR = 28.59 sleep disorders BMI ≥35	OR = 1.86 mental disorders BMI > 30 OR = 1.56 mental disorders BMI ≥ 35	53.3%, BMI > 30.0	OR = 1.05 BMI > 30 OR = 0.9 BMI ≥ 35	OR = 1.11 BMI > 30 OR = 0.7 BMI ≥ 35 habit forming/narcotic drugs	nd	nd
**Ulhôa et al., 2011— (Brazil) [8]**	42 male/TDs (39.8 ± 6.2 years)	cross-sectional	Sructured self-administered questionnaire, Actigraph, cortisol levels, cardiovascular blood parameters.	Actigraphic sleep duration (min) Day shifts: 393.5 ± 70.9 Irregular shifts: 410.7 ± 89.3	Job demand score: 16.9 mean Day Shift 14.1 mean Irregular Shift Minor psychiatric disorder score (SQR-20): 2.9 mean Day Shift 2.2 mean Irregular Shift	26.4 mean Day Shift 28.5 mean Irregular Shift	65.4% Day Shift 54.8% Irregular Shift	nd	nd	11.5% (on day shifts 16.1% (on irregular shifts)

nd: data not available; BMI: body mass index; PSQI: Pittsburg sleep questionnaire inventory; ESS: Epworth sleepiness scale GHQ-12; General health questionnaire; CFQ: Chalder Fatigue Questionnaire; AUDIT: Alcohol Use Disor- Identification Test Consumption; HEATS: Healthy Trucker Survey Instrument) questionnaire.

**Table 2:** Data summaries of the eight published papers included in the current analysis regards to risk of road accidents.

Author Year—(Country)	Risk of Road Accidents
de Oliveira LG et al., 2015—(Brazil) [55]	NE
Garbarino S et al., 2017—(Italy) [51]	TDs with insomnia driving accidents OR 1.82 (95% CI 1.33–2.49) near-miss accidents OR 3.35 (95% CI 2.06–5.45)
Guglielmi O et al., 2018—(Italy) [52]	NE
Meuleners L et al., 2015—(Australia) [54]	TDs with OSA driving accidents OR 3.42 (95% CI 1.34–8.72) TDs with depression driving accidents OR 6.59 (95% CI 1.30–33.24) TDs with not completed fatigue management driving accidents OR 6.05 (95% CI 1.80–20.24) TDs older drivers driving accidents OR 0.25 (95% CI 0.08–0.82)
Rosso et al., 2016—(Italy) [53]	TDs with age > 55 years old sudden-onset sleepiness at the wheel OR 5.22 (95% CI 1.29–21.1) TDs driving more than 50,000 km per year sudden-onset sleepiness at the wheel OR 2.89 (95% CI 1.37–6.11) TDs with Chalder Fatigue Questionnaire score > 11 sudden-onset sleepiness at the wheel OR 2.97 (95% CI 1.22–7.21)
Shattell M et al., 2012—(USA) [56]	NE
Thiese MS et al., 2015—(USA) [57]	NE
Ulhôa MA et al., 2011—(Brazil) [8]	NE

NE: not evaluated.
